# Association of Gonadotropin-Releasing Hormone Agonists for Prostate Cancer With Cardiovascular Disease Risk and Hypertension in Men With Diabetes

**DOI:** 10.1001/jamanetworkopen.2022.25600

**Published:** 2022-08-08

**Authors:** E. Lin, Hans Garmo, Mieke Van Hemelrijck, Björn Zethelius, Pär Stattin, Emil Hagström, Jan Adolfsson, Danielle Crawley

**Affiliations:** 1 School of Cancer and Pharmaceutical Sciences, Translational Oncology and Urology Research (TOUR), King’s College London, London, United Kingdom; 2 Department of Surgical Sciences, Uppsala University, Uppsala, Sweden; 3 Department of Public Health/Geriatrics, Uppsala University, Uppsala, Sweden; 4 Department of Medical Sciences, Uppsala University, Uppsala, Sweden; 5 Uppsala Clinical Research Centre, Uppsala, Sweden; 6 Department of Clinical Science, Intervention and Technology, Karolinska Institute, Stockholm, Sweden

## Abstract

**Importance:**

Men with type 2 diabetes have an increased risk of cardiovascular disease (CVD). Meanwhile, gonadotropin-releasing hormone (GnRH) agonists used in prostate cancer (PCa) are associated with increased risk of CVD.

**Objective:**

To evaluate the association between GnRH agonist use, PCa diagnosis per se, and CVD risk in men with type 2 diabetes.

**Design, Setting, and Participants:**

This nationwide population-based cohort study identified men with type 2 diabetes by use of data in the Prostate Cancer Data Base Sweden version 4.1 and the Swedish National Diabetes Register, with longitudinal data from 2006 to 2016. These data were used to create 2 cohorts, 1 including men with and without PCa and the other including men with PCa who received and did not receive GnRH agonists. Data analysis was conducted from January 2006 to December 2016.

**Exposures:**

Treatment with GnRH agonists and PCa diagnosis were the primary exposures.

**Main Outcomes and Measures:**

Primary outcome was a 10% increase in predicted 5-year CVD risk score. Secondary outcome was worsening hypertension as defined by the European Society of Hypertension Guidelines. Cox proportional hazards regression models were used to analyze the association.

**Results:**

The PCa exposure cohort included 5714 men (median [IQR] age, 72.0 [11.0]), and the non-PCa cohort included 28 445 men without PCa (median [IQR] age, 72.0 [11.0]). The GnRH agonist–exposure cohort included 692 men with PCa who received a GnRH agonist, compared with 3460 men with PCa who did not receive a GnRH agonist. Men with PCa receiving GnRH agonists had an increased estimated 5-year CVD risk score compared with men without PCa (hazard ratio [HR], 1.25; 95% CI, 1.16-1.36) and compared with men with PCa not receiving GnRH agonists (HR, 1.53; 95% CI, 1.35-1.74). Men receiving GnRH agonists had decreased blood pressure compared with men without PCa (HR, 0.70; 95% CI, 0.61-0.80) and compared with men with PCa not receiving GnRH agonists (HR, 0.68; 95% CI, 0.56-0.82).

**Conclusions and Relevance:**

In this population-based cohort study, there was an increased risk of CVD in men with type 2 diabetes who received a GnRH agonist for PCa. These findings highlight the need to closely control CVD risk factors in men with type 2 diabetes treated with GnRH agonists. The association between GnRH agonist use and decreased blood pressure levels warrants further study.

## Key Points

**Question:**

What is the risk of increased cardiovascular disease (CVD) risk in men with type 2 diabetes who receive gonadotropin-releasing hormone (GnRH) agonists for prostate cancer (PCa)?

**Findings:**

In this cohort study of 5714 men with PCa and 28 445 men without PCa, men with type 2 diabetes and PCa who received a GnRH agonist had 53% higher risk of an increase in CVD risk compared with men with type 2 diabetes and PCa who did not receive a GnRH agonist.

**Meaning:**

These findings suggest that there is a need to control CVD risk factors in men with type 2 diabetes who are treated with GnRH agonists.

## Teaser

This cohort study evaluates the association between gonadotropin-releasing hormone agonist use, prostate cancer diagnosis, and cardiovascular (CVD) risk in men with type 2 diabetes.

## Introduction

Prostate cancer (PCa) is one of the most prevalent cancers in men, with more than 1 million men diagnosed globally in 2019.^1^ Men with PCa have a higher risk of cardiovascular disease (CVD) than men without PCa.^2^ Intriguingly, CVD is the most common cause of death in men with PCa, accounting for 32%,^2^ while PCa accounts for just 20%.^3^

Gonadotropin-releasing hormone (GnRH) agonists are widely used in men with PCa.^3^ Use of GnRH agonists may cause several metabolic effects, including hypertension, dyslipidemia, and insulin resistance.^4^ These adverse effects are part of a metabolic-like syndrome that is a risk factor for CVD.^4,5^ An increased risk of CVD in men receiving GnRH agonists has consistently been demonstrated.^4,5^ Nevertheless, little evidence is available regarding the changes of CVD risk over time, which could inform and improve management of CVD risk factors, with an overall goal of reducing risk of CVD in men with PCa treated with a GnRH agonist.

Notably, GnRH agonist use is also associated with increased risk of type 2 diabetes .^4,6,7^ Type 2 diabetes is another important risk factor of CVD.

Previously, we have evaluated the association between GnRH agonist use and increased hemoglobin A_1c_ (HbA_1c_) levels and worsening control of lipid levels in men with preexisting type 2 diabetes (E. Lin, unpublished data, 2022).^8^ Increased risk of hypertension, another important risk factor for CVD, has been observed in men with PCa treated with GnRH agonists.^9^ However, data are inconsistent on the association between GnRH agonists and hypertension. For example, a cross-sectional study showed that hypertension was more prevalent in men receiving GnRH agonists than in age- and sex-matched persons not receiving GnRH agonists,^10^ whereas no significant change in blood pressure after 12 months of treatment with GnRH agonists was observed in another study.^11,12^

We conducted this nationwide population-based cohort study of men with preexisting type 2 diabetes aiming to evaluate the association of exposure to GnRH agonists and PCa diagnosis per se with CVD risk and worsening hypertension. In addition, we assessed changes in CVD risk over time, rather than CVD outcomes, to give clinically meaningful information to allow clinicians to focus on controlling CVD risk factors in men receiving GnRH agonists.

## Methods

The study was approved by the Research Ethics Board at Uppsala University, Sweden. As this study used data from established national registers, informed consent was not applicable for this study. The study followed the Strengthening the Reporting of Observational Studies in Epidemiology (STROBE) reporting guideline.

### Data Source

The study included men with type 2 diabetes registered in the Swedish National Diabetes Register (NDR) between January 1, 2006, and December 31, 2016. Information on PCa diagnosis for these men was obtained from the Prostate Cancer Data Base Sweden (PCBaSe), version 4.1.

NDR has enrolled 90% of all individuals with type 2 diabetes in Sweden since 1996 and collects data on outpatient visits,^13^ including longitudinal clinical characteristics on diabetes, hypertension, and hyperlipidemia (ie, HbA_1c_ levels, blood pressure, and lipid levels) as well as other clinical data (eg, albuminuria, atrial fibrillation, and a history of CVD).

The National Prostate Cancer Register (NPCR) of Sweden captures 98% of men diagnosed with PCa since 1998.^14^ The NPCR collects information on date of PCa diagnosis and PCa characteristics, such as stage of PCa and prostate-specific antigen (PSA) level. In PCBaSe, NPCR has been linked to other nationwide registers, including the Swedish Prescribed Drug Register (SPDR), National Patient Register, and a longitudinal database on socioeconomic factors (LISA) by use of the Swedish personal identification number. This linkage enabled us to obtain data on demographic characteristics, comorbidities, socioeconomic characteristics, and use of antihypertensive drugs assessed by filled prescriptions. PCBaSe also includes 5 randomly selected men without PCa for each individual with PCa, matched on birth year and county of residence.

### Study Population

We created 2 cohorts based on data for men with type 2 diabetes in the NDR and PCBaSe. The first cohort was the PCa-exposure cohort, including men diagnosed with PCa and receiving or not receiving GnRH agonists vs men without a PCa diagnosis. In this cohort, we aimed to explore the association of PCa diagnosis with CVD risk. This cohort also enabled us to estimate the association between use of GnRH agonists and CVD risk.

We further aimed to explore the association between life-long GnRH agonist use^15^ and CVD risk among men with type 2 diabetes and PCa without other coadministered treatments for PCa. Thus, we created the GnRH agonist–exposure cohort, including men with PCa receiving GnRH agonists compared with men with PCa not receiving GnRH agonists (eFigure 1 in the Supplement). In particular, we excluded men who received GnRH agonists neoadjuvantly and adjuvantly to radical radiotherapy, given that these men were exposed to GnRH agonists for a limited time.

The PCa-exposure cohort included men who had at least 4 dates of registration in NDR and were diagnosed with PCa after their third registered date. The start of follow-up was the date of PCa diagnosis. In the GnRH agonist–exposure cohort, we selected men who were diagnosed with PCa and treated with GnRH agonists after the third date of NDR registration. The start of follow-up was the date of the first filled prescription for a GnRH agonist in the SPDR. For both cohorts, we selected 5 men without the exposure for each man with the exposure from the NDR, matched on the number of NDR registrations and average duration between NDR visits for each man with exposure. Start of follow-up for a man without exposure was inherited from the corresponding man with exposure (eFigure 2 in the Supplement).

Data on baseline characteristics in both cohorts were collected from the 3 last NDR records prior to the start of follow-up to reduce misclassification bias at the baseline (eFigure 2 in the Supplement). In the NDR, approximately 3% to 6% of men had missing data of a baseline measurement. We used the last observation carried forward method to substitute these missing data. For instance, if the last observation for each variable of interest in the NDR was missing, the information was retrieved from the second to last observation. If all 3 last NDR observations were missing, we classified the data as missing.

The baseline characteristics are presented in Table 1. *P* values were not estimated because of a lack of a null hypothesis that men treated with GnRH agonists and not treated with GnRH agonists should be equal given matching.^16,17,18^

**Table 1.** Baseline Characteristics of Men With Type 2 Diabetes in the Swedish National Diabetes Register Diagnosed With PCa Receiving and Not Receiving GnRH Agonists Between 2006 and 2016 and Their Matched Counterparts

Baseline characteristics	Exposure cohort, No. (%)			
	PCa		GnRH agonists	
	Men with PCa (n = 5714)	Men without PCa (n = 28 445)	Men receiving GnRH agonists (n = 692)	Men not receiving GnRH agonists (n = 3460)
Patient characteristics				
Age, median (IQR)	72.0 (67.0-78.0)	73.0 (68.0-79.0)	78.0 (72.0-83.0)	74.0 (69.8-79.3)
Education level				
Low	2340 (41.0)	12 231 (43.0)	308 (44.5)	1333 (38.5)
Middle	2755 (48.2)	13 172 (46.3)	297 (42.9)	1678 (48.5)
High	570 (10.0)	2686 (9.4)	82 (11.8)	432 (12.5)
Missing	49 (0.9)	356 (1.3)	5 (0.7)	17 (0.5)
Civil status				
Married	3708 (64.9)	17 582 (61.8)	444 (64.2)	2248 (65.0)
Not married, ie, divorced, widowed, or missing	2006 (35.1)	10 863 (38.2)	248 (35.8)	1212 (35.0)
CCI				
0	2625 (45.9)	11 253 (39.6)	225 (32.5)	1062 (30.7)
1	1559 (27.3)	7853 (27.6)	210 (30.3)	1168 (33.8)
2	673 (11.8)	3739 (13.1)	94 (13.6)	498 (14.4)
≥3	857 (15.0)	5600 (19.7)	163 (23.6)	732 (21.2)
Smoking				
No	4581 (80.2)	22 273 (78.3)	524 (75.7)	2683 (77.5)
Yes	553 (9.7)	2912 (10.2)	51 (7.4)	267 (7.7)
Missing	580 (10.2)	3260 (11.5)	117 (16.9)	510 (14.7)
Physical activity^a^				
Daily	573 (10.0)	3452 (12.1)	98 (14.2)	380 (11.0)
3-5 times a week	500 (8.8)	2486 (8.7)	69 (10.0)	268 (7.7)
1-2 times a week	902 (15.8)	4261 (15.0)	96 (13.9)	503 (14.5)
Less than once a week	1016 (17.8)	4786 (16.8)	104 (15.0)	629 (18.2)
Never	1531 (26.8)	6919 (24.3)	140 (20.2)	784 (22.7)
Missing	1192 (20.9)	6541 (23.0)	185 (26.7)	896 (25.9)
BMI, median (IQR)	28.3 (25.7-31.2)	28.4 (25.8-31.5)	28.2 (25.8-31.1)	27.9 (25.5-30.9)
No. of clinic visits				
3 to <10	3794 (66.4)	18 879 (66.4)	504 (72.8)	2520 (72.8)
10 to <20	1520 (26.6)	7578 (26.6)	151 (21.8)	755 (21.8)
20 to <30	315 (5.5)	1567 (5.5)	29 (4.2)	145 (4.2)
≥30	85 (1.5)	421 (1.5)	8 (1.2)	40 (1.2)
PCa status				
PCa diagnosis				
No PCa	0	28 445 (100)	0	0
PCa	5714 (100)	0	692 (100)	3460 (100)
Receipt of GnRH agonists				
No PCa	0	28 445 (100)	0	0
No	4274 (74.8)	0	0	3460 (100)
Yes	1400 (25.2)	0	692 (100)	0
PCa risk category				
No PCa	0	28 445 (100)	0	0
Low	1122 (19.8)	0	145 (21.0)	1437 (41.5)
Intermediate	1838 (32.2)	0	229 (33.1)	1272 (36.8)
High	1531 (26.8)	0	232 (33.5)	533 (15.4)
Metastases				
Regional	389 (6.8)	0	42 (6.1)	56 (1.6)
Distance	650 (11.4)	0	32 (4.6)	39 (91.1)
Missing data	184 (3.2)	0	12 (1.7)	123 (3.6)
Type 2 diabetes status				
Duration, y				
<10	2751 (48.1)	12 755 (44.8)	320 (46.2)	1703 (49.2)
10 to <20	1939 (33.9)	10 149 (35.7)	222 (32.1)	1139 (32.9)
20 to <30	530 (9.3)	3123 (11.0)	78 (11.3)	310 (9.0)
≥30	158 (2.8)	921 (3.2)	23 (3.3)	90 (2.6)
Missing	336 (5.9)	1497 (5.3)	49 (7.1)	218 (6.3)
HbA_1c_ level, median (IQR), mmol/mol	51.0 (45.0-59.0)	53.0 (46.0-62.0)	51.0 (45.0-58.5)	51.0 (45.0-60.0)
Primary type 2 diabetes treatment				
Diet	1333 (23.3)	6196 (21.8)	195 (28.2)	867 (25.1)
Oral hypoglycemic drugs^b^	2513 (44.0)	12 003 (42.2)	260 (37.6)	1513 (43.7)
Insulin	1868 (32.7)	10 246 (36.0)	237 (34.2)	1080 (31.2)
Hypertension status				
SBP, median (IQR), mmHg	135.0 (125.0-145.0)	135.0 (125.0-145.0)	134.0 (125.0-142.0)	135.0 (125.0-145.0)
DBP, median (IQR), mmHg	77.0 (70.0-80.0)	75.0 (70.0-80.0)	72.0 (68.0-80.0)	75.0 (70.0-80.0)
No. of BP drugs				
0	867 (15.2)	4212 (14.8)	110 (15.9)	496 (14.3)
1	1353 (23.7)	6674 (23.5)	164 (23.7)	911 (26.3)
2	1702 (29.8)	8308 (29.2)	198 (28.6)	1007 (29.1)
3	1300 (22.8)	6727 (23.6)	176 (25.4)	794 (22.9)
≥4	492 (8.6)	2524 (8.8)	44 (6.3)	252 (7.3)
Hyperlipidemia				
Non–HDL-C level, median (IQR), mmol/L	3.1 (2.5-3.8)	3.1 (2.5-3.8)	3.0 (2.5-3.7)	3.0 (2.5-3.8)
Total cholesterol, median (IQR), mmol/L	4.3 (3.7-5.0)	4.3 (3.7-5.0)	4.4 (3.8-5.0)	4.4 (3.8-5.1)
Triglyceride, median (IQR), mmol/L	1.4 (1.0-2.0)	1.5 (1.1-2.1)	1.3 (1.0-1.8)	1.4 (1.0-2.0)
LDL-C, median (IQR), mmol/L	2.4 (1.9-3.0)	2.3 (1.8-3.0)	2.3 (1.9-3.0)	2.4 (1.9-3.0)
HDL-C, median (IQR), mmol/L	1.1 (1.0-1.4)	1.1 (1.0-1.4)	1.2 (1.0-1.5)	1.2 (1.0-1.4)
CVD risk status				
5-y CVD risk score, median (IQR), %	11.4 (11.1-12.0)	11.4 (11.0-11.9)	11.7 (11.3-12.2)	11.4 (11.1-12.0)

Abbreviations: BMI, body mass index (calculated as weight in kilograms divided by height in meters squared); BP, blood pressure; CCI, Charlson Comorbidity Index; DBP, diastolic blood pressure; GnRH, gonadotropin-releasing hormone; HbA_1c_, hemoglobin A_1c_; HDL-C, high-density lipoprotein cholesterol; LDL-C, low-density lipoprotein cholesterol; PCa, prostate cancer; SBP, systolic blood pressure.

^a^ In the National Diabetes Register, physical activity is defined as at least 30 minutes of walking or a similar activity.

^b^ The classification of antihyperglycemic medication was based on the efficacy of hyperglycemic medications in blood glucose level control and safety profiles, as well as the severity of type 2 diabetes in men in our study.

### Exposure

Treatment with GnRH agonists was the primary exposure in our study. Additionally, we also aimed to explore the association of PCa diagnosis and PCa risk categories with CVD risk and hypertension. Based on a modification of the National Comprehensive Cancer Network guideline used in NPCR,^19^ men with PCa were classified into 5 PCa risk categories. Low risk was defined as stage T1 or T2a, with a PSA level of less than 10 ng/mL (to convert to micrograms per liter, multiply by 1.0) and Gleason score of 6. Intermediate risk was defined as stage T2b or T2c, with a PSA level of less than 20 ng/mL or Gleason score 7. High risk was defined as stage T3a or T4, with PSA level of 20 ng/mL or greater or Gleason score ≥8. Regional metastases were defined as any T, N1, and M0 stage. Distant metastases were defined as any T or N, and M1 stage.

### Outcome

The primary outcomes of the study were 10% or 5% increase in predicted 5-year CVD risk score, which was estimated by use of a CVD risk model. The CVD risk model was devised by Zethelius et al^20^ using data from the NDR, the same data source used in this study. The risk model was based on 12 factors, including age, gender, diabetes duration, HbA_1c_ level, systolic blood pressure, total-to–high-density lipoprotein cholesterol ratio, weight, height, smoking, albuminuria, atrial fibrillation, and a history of CVD.^20^ This 5-year risk score has been validated in the same data source of our study, with sufficient calibration (ratio of estimated 4-year risk to observed rate, 0.97) and discrimination (for top quartile: *C* statistic, 0.72; sensitivity, 51%; and specificity, 78%). When we calculated the estimated 5-year CVD risk score, we used last observation carried forward to impute these missing data. If missing data remained after last observational carried forward, we further used mean imputation. The mean value used in the imputation was based on the NDR report, which represents the population mean value for each variable in the NDR.^21^

We also investigated the association between use of GnRH agonists and PCa diagnosis and hypertension. We defined the outcome based on the European Society of Hypertension guidelines,^22^ as follows: systolic blood pressure of 140 mm Hg or greater and diastolic blood pressure of 80 mm Hg or greater in men with type 2 diabetes older than 65 years or systolic blood pressure of 130 mm Hg or greater and diastolic blood pressure of 80 mm Hg or greater in men with type 2 diabetes aged 65 years or younger; 10–mm Hg increase in systolic blood pressure; 5–mm Hg increase in diastolic blood pressure; dose escalation in different antihypertensive drug monotherapies; and antihypertensive drug started or an escalation of antihypertensive treatment by sequentially adding other classes of antihypertensive drug. All outcomes in our study were defined as the first event occurred since the start of follow-up.

### Statistical Analysis

Cox proportional hazards regression models were used to estimate hazard ratios (HRs) and 95% CIs for the increased estimated 5-year CVD risk score and elevated blood pressure. In the adjusted models to evaluate the HR and 95% CIs of increased CVD risk, we adjusted for physical activity, educational level, civil status, and Charlson Comorbidity Index (CCI), excluding cardiovascular diseases. When we estimated the HR and 95% CIs of worsening hypertension, the models were adjusted for age at PCa diagnosis, physical activity, smoking, body mass index (BMI), HbA_1c_ level, diabetes medication, duration of diabetes, educational level, civil status, CCI, average duration of NDR visits, and number of NDR registrations. PCa was another study exposure, and hence PCa risk category was not adjusted for in the final model. While several traditional risk factors are well known, such as physical activity, metabolic risk factors, and comorbidities, other risk factors, such as socioeconomic factors (ie, education level and marital status), are increasingly being recognized. Several studies have reported the association between education level and marital status and incidence of CVD and PCa, adherence to GnRH agonists, and adverse outcomes for CVD and PCa.^15,23,24,25,26,27^ Therefore, education level and marital status were considered as confounders, and we adjusted for them in our analyses.

Kaplan-Meier curves were used to present cumulative incidence of the increase in estimated 5-year CVD risk score and elevated blood pressure over time. Line graphs were used to illustrate measurement changes for blood pressure levels over time. The mean value of blood pressure levels in the line graph for all men was calculated every 3 months from 6 months before the start of follow-up to 2 years after the start of the follow-up. The mean value was determined through linear interpolation of the 2 adjacent blood pressure level values assuming a linear relationship between 2 consecutive values. The 2 adjacent values were on both sides of the time point of every 3 months from the start of follow-up. All statistical analyses were performed using R version 3.5.2 (R Foundation for Statistical Computing) and SAS version 9.4 (SAS Institute).

## Results

The PCa exposure cohort included 5714 men (median [IQR] age, 72 [11.0]) diagnosed with PCa and 28 445 men (median [IQR] age, 72 [11.0]) without PCa, whereas the GnRH agonist–exposure cohort included 692 men with PCa and receiving GnRH agonists and 3460 men with PCa but not receiving GnRH agonists. Both groups in each cohort had similar baseline characteristics (Table 1).

### CVD Risk

#### PCa-Exposure Cohort

We found an increased predicted 5-year CVD risk score in men with PCa receiving GnRH agonists (10% increase in the score: HR, 1.25; 95% CI, 1.16-1.36) (Table 2; eTable 2 in the Supplement). There was an increase in predicted 5-year CVD risk score in men diagnosed with PCa regardless of receipt of GnRH agonists compared with men without PCa with type 2 diabetes (10% increase in the score: HR, 1.07; 95% CI, 1.03-1.12) (Table 2; eTable 2 in the Supplement). When grouped into risk categories, the increased CVD risk was only found in men with regional or distant metastatic disease (Table 2).

**Table 2.** HRs and 95% CIs for Increased CVD Risk by Using Different Definitions of Events in PCa-Exposure Cohort and GnRH Agonist–Exposure Cohort

Exposure	HR (95% CI)			
	10% increase in 5-y CVD risk score		5% increase in 5-y CVD risk score	
	Crude model	Adjusted model^a^	Crude model	Adjusted model^a^
**PCa-exposure cohort**				
Using GnRH agonists				
No PCa	1 [Reference]	1 [Reference]	1 [Reference]	1 [Reference]
PCa without GnRH agonists	1.06 (1.01-1.11)	1.02 (0.98-1.07)	1.02 (0.98-1.06)	0.98 (0.94-1.02)
PCa with GnRH agonists	1.23 (1.14-1.33)	1.25 (1.16-1.36)	1.13 (1.05-1.21)	1.12 (1.05-1.20)
PCa diagnosis				
No PCa	1 [Reference]	1 [Reference]	1 [Reference]	1 [Reference]
PCa	1.10 (1.05-1.14)	1.07 (1.03-1.12)	1.04 (1.00-1.08)	1.01 (0.98-1.05)
PCa risk category				
No PCa	1 [Reference]	1 [Reference]	1 [Reference]	1 [Reference]
Low	1.04 (0.96-1.13)	0.98 (0.90-1.07)	1.02 (0.95-1.10)	0.97 (0.91-1.05)
Intermediate	1.10 (1.02-1.17)	1.06 (0.99-1.13)	1.04 (0.99-1.11)	1.01 (0.96-1.07)
High	1.04 (0.96-1.12)	1.05 (0.97-1.13)	0.99 (0.92-1.05)	0.98 (0.92-1.05)
Metastases				
Regional	1.18 (1.01-1.38)	1.23 (1.06-1.44)	1.10 (0.96-1.26)	1.10 (0.96-1.26)
Distant	1.31 (1.16-1.49)	1.25 (1.10-1.42)	1.17 (1.05-1.30)	1.11 (1.00-1.24)
Missing data	1.31 (1.06-1.61)	1.34 (1.09-1.65)	1.12 (0.94-1.34)	1.15 (0.96-1.38)
**GnRH agonist–exposure cohort**				
Using GnRH agonists				
PCa not using GnRH agonists	1 [Reference]	1 [Reference]	1 [Reference]	1 [Reference]
PCa using GnRH agonists	1.49 (1.32-1.69)	1.53 (1.35-1.74)	1.24 (1.11-1.37)	1.27 (1.13-1.42)
PCa risk category				
Low	1 [Reference]	1 [Reference]	1 [Reference]	1 [Reference]
Intermediate	1.02 (0.91-1.14)	1.02 (0.91-1.14)	1.06 (0.97-1.16)	1.05 (0.96-1.16)
High	1.02 (0.89-1.17)	1.01 (0.87-1.16)	0.98 (0.88-1.10)	0.95 (0.85-1.07)
Metastasises				
Regional	0.83 (0.58-1.17)	0.96 (0.67-1.38)	0.96 (0.74-1.26)	1.04 (0.79-1.36)
Distant	1.13 (0.77-1.64)	1.10 (0.75-1.62)	1.01 (0.73-1.40)	1.08 (0.77-1.50)
Missing data	0.91 (0.67-1.23)	0.87 (0.64-1.19)	0.98 (0.78-1.25)	0.92 (0.72-1.18)

Abbreviations: CVD, cardiovascular diseases; HR, hazard ratio; GnRH, Gonadotropin-releasing hormone; PCa, prostate cancer.

^a^ In the adjusted models used to evaluate the HR and 95% CI of increased CVD risk, we adjusted for age at PCa diagnosis, body mass index, smoking, physical activity, educational level, civil status, lipid levels (low-density lipoprotein, triglyceride, total cholesterol, and non–high-density lipoprotein), diabetes status (hemoglobin A_1c_ level, duration of type 2 diabetes, and antidiabetic drugs), blood pressure levels (systolic blood pressure level and diastolic bold pressure level), Charlson Comorbidity Index (excluding cardiovascular diseases), average of duration between National Diabetes Register visits and number of National Diabetes Register registrations.

**Figure 1.**
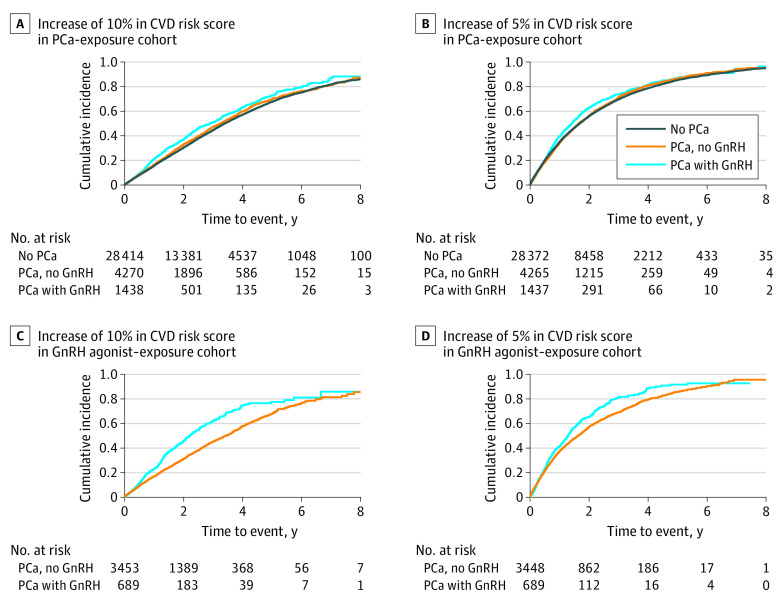
Cumulative Incidence of Cardiovascular Disease (CVD) Risk by Prostate Cancer (PCa) Status and Exposure to Gonadotropin-Releasing Hormone (GnRH) Agonists A and B, Men receiving GnRH agonists in the PCa-exposure cohort had a higher cumulative incidence of increased CVD risk compared with men without PCa. This was more apparent when there was a 10% increase in 5-year CVD risk (A). C and D, Men with PCa receiving GnRH agonists had a higher cumulative incidence of increased CVD than men with PCa not receiving GnRH agonists.

Figure 1A and B shows a higher cumulative incidence for increased predicted 5-year CVD risk score in men receiving GnRH agonists than in men not receiving GnRH agonists. The Kaplan-Meier curves also showed the trend of cumulative incidence change over time, and we found that the largest difference in cumulative incidence of the increased risk score between men with PCa regardless of receipt of GnRH agonists after 3 years of exposure.

#### GnRH Agonist–Exposure Cohort

We observed an association between use of GnRH agonists and increased predicted 5-year CVD risk score in men with PCa receiving GnRH agonists compared with men with PCa not receiving GnRH agonists (10% increase in the score: HR, 1.53; 95% CI, 1.35-1.74) (Table 2; eTable 2 in the Supplement). Figure 1C and D shows that men with PCa receiving GnRH agonists had a higher cumulative incidence of an increased risk score over time compared with men with PCa not receiving GnRH agonists.

### Blood Pressure

#### PCa-Exposure Cohort

We found lower blood pressure in men diagnosed with PCa regardless of receipt of GnRH agonists in the adjusted model (HR, 0.81; 95% CI, 0.76-0.87) (Table 3; eTable 1 and eTable 2 in the Supplement). However, no association was observed between antihypertensive drug escalation and use of GnRH agonists or PCa diagnosis (Table 3; eTable 1 and eTable 2 in the Supplement). Moreover, a decreased risk of elevated blood pressure was observed for men in all PCa risk categories, except for a 10–mm Hg increase in systolic blood pressure (Table 3; eTable 1 in the Supplement).

**Table 3.** Adjusted HRs and 95% CIs for Worsening Hypertension Using Different Definitions of Events in PCa-Exposure Cohort and GnRH Agonist–Exposure Cohort^a^

Exposure	HR (95% CI)					
	BP increased^c^	Increased			Anti-hypertensive drug changes^b^	
		SBP 10 mm Hg^d^		DBP 5 mm Hg^e^	No.	Dosage
**PCa-exposure cohort**						
Using GnRH agonists						
No PCa	1 [Reference]	1 [Reference]	1 [Reference]		1 [Reference]	1 [Reference]
PCa without GnRH agonists	0.85 (0.79-0.91)	0.94 (0.89-0.98)	0.89 (0.85-0.94)		0.97 (0.92-1.03)	1.00 (0.95-1.05)
PCa with GnRH agonists	0.70 (0.61-0.80)	0.81 (0.74-0.88)	0.76 (0.70-0.84)		0.95 (0.86-1.04)	0.99 (0.91-1.08)
PCa diagnosis						
No	1 [Reference]	1 [Reference]	1 [Reference]		1 [Reference]	1 [Reference]
Yes	0.81 (0.76-0.87)	0.90 (0.86-0.95)	0.86 (0.82-0.90)		0.97 (0.92-1.02)	1.00 (0.95-1.04)
PCa risk category						
No PCa	1 [Reference]	1 [Reference]	1 [Reference]		1 [Reference]	1 [Reference]
Low	0.96 (0.85-1.09)	0.97 (0.89-1.06)	0.94 (0.86-1.03)		0.98 (0.90-1.08)	1.00 (0.91-1.09)
Intermediate	0.81 (0.72-0.90)	0.90 (0.84-0.97)	0.88 (0.82-0.95)		1.04 (0.97-1.12)	1.06 (0.99-1.14)
High	0.78 (0.69-0.88)	0.85 (0.78-0.92)	0.79 (0.73-0.86)		0.96 (0.88-1.05)	1.01 (0.93-1.09)
Metastases						
Regional	0.71 (0.54-0.92)	0.92 (0.78-1.08)	0.76 (0.64-0.90)		0.82 (0.68-1.00)	0.87 (0.73-1.03)
Distant	0.66 (0.54-0.81)	0.89 (0.77-1.02)	0.85 (0.74-0.97)		0.77 (0.65-0.90)	0.79 (0.68-0.92)
Missing data	0.90 (0.64-1.24)	1.01 (0.81-1.26)	0.95 (0.77-1.18)		0.98 (0.77-1.25)	1.08 (0.87-1.34)
**GnRH Agonist–exposure cohort**						
Using GnRH agonists						
PCa without GnRH agonist	1 [Reference]	1 [Reference]	1 [Reference]		1 [Reference]	1 [Reference]
PCa with GnRH agonist	0.68 (0.56-0.82)	0.95 (0.83-1.08)	0.94 (0.83-1.07)		1.03 (0.90-1.19)	1.03 (0.90-1.19)
PCa risk group						
Low	1 [Reference]	1 [Reference]	1 [Reference]		1 [Reference]	1 [Reference]
Intermediate	1.03 (0.87-1.21)	0.98 (0.88-1.09)	1.00 (0.90-1.12)		1.05 (0.93-1.17)	1.05 (0.93-1.17)
High	0.93 (0.77-1.13)	0.85 (0.73-0.97)	1.00 (0.87-1.14)		1.04 (0.90-1.20)	1.04 (0.90-1.20)
Metastases						
Regional	0.86 (0.51-1.47)	0.65 (0.45-0.94)	0.65 (0.45-0.94)		1.18 (0.86-1.63)	1.18 (0.86-1.63)
Distant	0.99 (0.55-1.79)	0.48 (0.29-0.80)	0.78 (0.52-1.17)		0.75 (0.47-1.18)	0.75 (0.48-1.18)
Missing data	0.95 (0.60-1.50)	0.89 (0.67-1.19)	0.78 (0.58-1.05)		1.19 (0.89-1.58)	1.19 (0.89-1.58)

Abbreviations: BP, blood pressure; DBP, diastolic blood pressure; GnRH, gonadotropin-releasing hormone; HR, hazard ratio; PCa, prostate cancer; SBP, systolic blood pressure.

^a^ Models for HR and 95% CI of worsening hypertension were adjusted for age at PCa diagnosis, body mass index, smoking, physical activity, educational level, civil status, lipid levels, diabetes status, and Charlson Comorbidity Index.

^b^ Men with missing data on antihypertensive drugs were excluded.

^c^ Men with missing data on SBP and DBP were excluded. Outcome defined as SBP increased to 140 mm Hg or greater (or 130 mm Hg or greater for men aged 65 years and younger) and DBP increased to 80 mm Hg.

^d^ Men with missing data on SBP were excluded.

^e^ Men with missing data on DBP were excluded.

**Figure 2.**
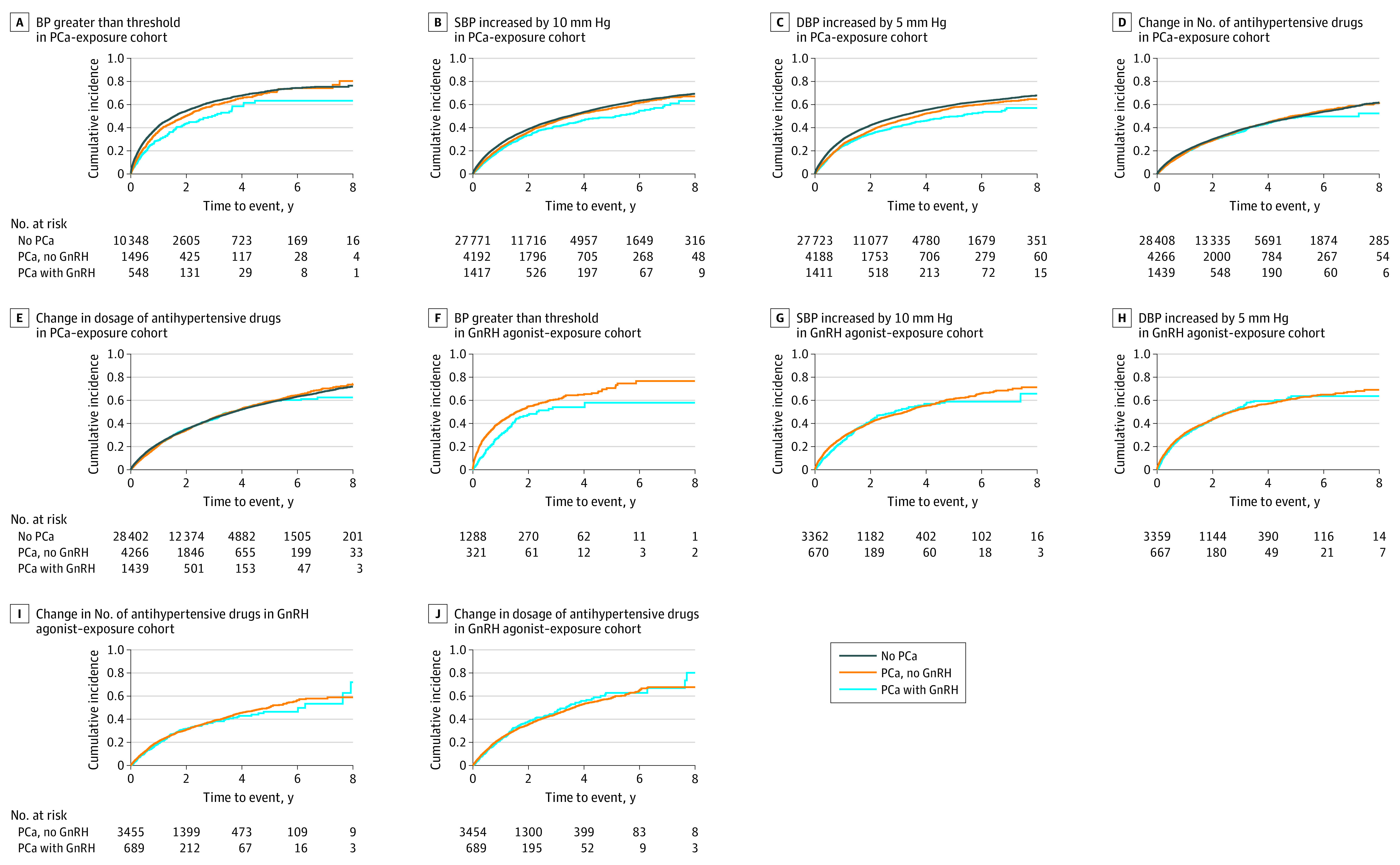
Cumulative Incidence of Worsening Hypertension by Prostate Cancer (PCa) Status and Exposure to Gonadotropin-Releasing Hormone (GnRH) Agonists A, Men with PCa receiving GnRH agonists had a lower cumulative incidence of systolic blood pressure (SBP) of 140 mm Hg or greater (or 130 mm Hg or greater for men aged 65 years or younger) and diastolic blood pressure (DBP) of 80 mm Hg compared with men without PCa. B and C, Men receiving GnRH agonists had a lower cumulative incidence of increases in SBP and DBP compared with men without PCa. D and E, No difference was seen between men receiving GnRH agonists and men without PCa in the cumulative incidence for the escalation of antihypertensive drugs. F, Men with PCa receiving GnRH agonists had a lower cumulative incidence of SBP increasing to 140 mm Hg (or 130 mm Hg for men aged 65 years or younger) and DBP increasing to 80 mm Hg compared with men with PCa not receiving GnRH agonists. G-J, No difference was seen between men with PCa receiving GnRHs and men with PCa not receiving GnRH agonists without PCa in the cumulative incidence for increased SBP (G), increasing DBP (H), and the escalation of antihypertensive drugs (I and J).

Men receiving GnRH agonists had a lower cumulative incidence of elevated blood pressure compared with men without PCa but similar cumulative incidence for the escalation of antihypertensive drugs (Figure 2A-E). Blood pressure levels in men with PCa receiving GnRH agonists were lower than for men not receiving GnRH agonists (eFigure 3 in the Supplement).

#### GnRH Agonist–Exposure Cohort

In this cohort, men with PCa receiving GnRH agonists had a decreased risk of elevated blood pressure compared with men with PCa not receiving GnRH agonists (HR, 0.68; 95% CI, 0.56-0.82) (Table 3 and Figure 2F-J; eTable 1 and eTable 2 in the Supplement). However, no association was found between use of GnRH agonists and other outcomes (Table 3; eTable 1, eTable 2 in the Supplement; Figure 2F-J). We observed a decreased risk of elevated systolic blood pressure in men with high-risk PCa, regional metastases, and distant metastases (Table 3; eTable 1 in the Supplement). The blood pressure levels in these men were always lower than in men with PCa not receiving GnRH agonists (eFigure 4 in the Supplement).

## Discussion

In this nationwide, population-based cohort study of CVD risk in more than 30 000 men with type 2 diabetes with 11 years follow-up, men receiving GnRH agonists had an increased predicted 5-year CVD risk score. We also found a decrease in blood pressure in men receiving GnRH agonists and in men with PCa.

### CVD Risk

Our study used the CVD risk score created and used by the NDR to predict the CVD risk changes over time in men with type 2 diabetes. The baseline characteristics, including risk factors for CVD, such as metabolic status, age, and smoking, were similar in both cohorts (Table 1). Men with type 2 diabetes receiving GnRH agonists had a 26% higher risk of a 10% increase in predicted 5-year CVD risk score during the 11-year follow-up compared with men with type 2 diabetes without PCa; men with type 2 diabetes and PCa receiving GnRH agonists had a 52% higher risk compared with men with type 2 diabetes and PCa not receiving GnRH agonists. The increase in predicted 5-year CVD risk score with GnRH agonist use was evident after 1 year of exposure in men with type 2 diabetes. The temporal nature of increased risk in men with type 2 diabetes and PCa receiving GnRH agonists warrants further investigation.

Several studies have previously reported on the association of use of GnRH agonists with CVD risk and mortality, although not in men with type 2 diabetes in particular.^7,28,29^ Based on these studies, the American Heart Association, along with the American Cancer Society and the American Urological Association, published a joint statement highlighting an association between use of GnRH agonists and increased risk of CVD.^30^

During the last decade, several studies have evaluated the association between GnRH agonists and increased risk of CVD.^4,31,32,33,34^ A study conducted by Keating et al^7^ reported that use of GnRH agonists was associated with 20% increased risk of incident coronary heart disease. In 2020, a meta-analysis^4^ reported an association between GnRH agonist use and acute myocardial infarction, with a risk ratio of 1.73 (95% CI, 1.05-2.85), as well as coronary heart disease, with a risk ratio of 2.09 (95% CI, 1.02-4.30). These associations are supported by underlying biological mechanisms including a direct effect on the activation of monocytes and T lymphocytes in the immune system and indirect effects, such as reducing circulating testosterone.^3,4,35^

The association between use of GnRH agonists and increased risk of type 2 diabetes has also been established and is also supported by several proposed underlying mechanisms, such as the low testosterone level induced by GnRH agonists.^11,12^ The US Food and Drug Administration issued a safety warning, requiring labeling on GnRH agonists warning of the increased risk of diabetes.^36^ Type 2 diabetes is associated with an increased risk of CVD and related mortality.^37^ Thus, GnRH agonists need to be used with caution in men with type 2 diabetes. Our findings expand on previous published studies showing the association between GnRH agonists and CVD risk, particularly in men with type 2 diabetes. The estimated 5-year CVD risk score used in our study also allows more insights into the changes of CVD risk over time through risk factors for CVD in the risk model. Our findings emphasize the need to monitor and control CVD risk factors, such as elevated blood glucose, abnormal lipid levels, and changes in kidney function (ie, albuminuria, estimated glomerular filtration rate, and serum creatinine levels), in men with type 2 diabetes and PCa who are receiving GnRH agonists. Additionally, after evaluating how CVD risk changes over time in men with type 2 diabetes and PCa treated with GnRH agonists as well as the association between metabolic risk factors for CVD risk and use of GnRH agonists, we propose that future research needs to further identify the proportion of indirect effects of these metabolic risk factors through GnRH agonists in men with type 2 diabetes by mediation analysis.

### Blood Pressure

Hypertension is an important CVD risk factor.^38^ However, few studies have assessed the association between use of GnRH agonists and hypertension. GnRH agonist use may be associated with elevated blood pressure because of its reduction in testosterone.^39^ However, several observational studies reported no change in blood pressure after 12 months of treatment with GnRH agonists.^11,12,40^ Testosterone has been shown to activate both vasodilator and vasoconstrictor pathways, which may explain the inconsistent results.^38,41^ In our study, use of GnRH agonists and PCa diagnosis were associated with lower blood pressure in men with type 2 diabetes. Our finding is in line with what could be expected from the results in preclinical study animal models that showed that castration decreases blood pressure.^41^ The inconsistent results on the association between GnRH agonist use, PCa diagnosis, and hypertension warrant further studies.

### Strengths and Limitations

To our knowledge this is one of the largest long-term nationwide population-based cohort studies examining the association between GnRH agonists and CVD risk in men with type 2 diabetes. We also included comprehensive data on patient characteristics, which enabled us to analyze 12 CVD risk factors to calculate the 5-year CVD risk score and estimate the CVD risk change over time. These 12 factors included metabolic disorders, kidney function, and CVD history, which are important to improve the performance of the CVD risk score in men with type 2 diabetes.^42^ When validating the 5-year risk model, the end point was fatal and nonfatal CVD (defined as the composite of chronic heart disease or stroke, whichever came first), which are more relevant for individuals with type 2 diabetes.^42^ Importantly, the risk score used in our study was created using data in the NDR and has been validated in the population from the same data source, showing sufficient calibration and discrimination, indicating an accurate estimation of CVD risk in our study. Therefore, there is no issue of generalizability of the risk score. These approaches enabled us to estimate the CVD risk changes over time accurately. Finally, by matching exposed and unexposed men on the number of NDR visits and average time between two NDR visits, we reduced the impact of health care–seeking behaviors, which are likely to be correlated with patient adherence and the quality of care.

This study has limitations as well. There may be residual confounding, as no information was available for variables including family history of hyperlipidemia and PCa. Another possible source of residual confounding was confounding by indication for GnRH agonist use. However, baseline characteristics of risk factors for CVD were similar between men receiving and not receiving GnRH agonists, indicating that the use of GnRH agonists was not affected by CVD risk factors. Because of the lack of data on CVD-related mortality, the association of GnRH agonists and PCa diagnosis with CVD death could not be studied.

## Conclusions

In this population-based cohort study, there was an increased risk of CVD in men with type 2 diabetes receiving GnRH agonists for PCa. Our findings highlight the need to monitor and control CVD risk factors in men with type 2 diabetes and PCa who are receiving GnRH agonists. In addition, we found a decreased risk of hypertension in men receiving GnRH agonists, which warrants further study.

## Notes

Supplement. **eTable 1. **HR and 95% CI for Worsening Hypertension Using Different Definitions of Events in PCa Exposure Cohort and GnRH Exposure Cohort: Crude Model **eTable 2. **Number of Events in PCa Cohort and GnRH Cohort **eFigure 1. **Patient Inclusion and Exclusion Flowchart **eFigure 2. **Schematic Diagram for the Selection Process of Exposed Men and Nonexposed Men in NDR Registrations **eFigure 3. **Blood Pressure Levels Change Over Time by PCa Status in PCa Exposure Cohort **eFigure 4. **Blood Pressure Levels Change Over Time by PCa Status in GnRH Exposure Cohorts

## References

[1] Rawla P. Epidemiology of prostate cancer. World J Oncol. 2019;10(2):63-89. doi:10.14740/wjon1191 31068988 PMC6497009

[2] Sturgeon KM, Deng L, Bluethmann SM, . A population-based study of cardiovascular disease mortality risk in US cancer patients. Eur Heart J. 2019;40(48):3889-3897. doi:10.1093/eurheartj/ehz766 31761945 PMC6925383

[3] Challa AA, Calaway AC, Cullen J, . Cardiovascular toxicities of androgen deprivation therapy. Curr Treat Options Oncol. 2021;22(6):47. doi:10.1007/s11864-021-00846-z 33866442 PMC8053026

[4] Liang Z, Zhu J, Chen L, . Is androgen deprivation therapy for prostate cancer associated with cardiovascular disease? a meta-analysis and systematic review. Andrology. 2020;8(3):559-574. doi:10.1111/andr.12731 31743594

[5] Van Hemelrijck M, Garmo H, Holmberg L, . Absolute and relative risk of cardiovascular disease in men with prostate cancer: results from the Population-Based PCBaSe Sweden. J Clin Oncol. 2010;28(21):3448-3456. doi:10.1200/JCO.2010.29.1567 20567006

[6] Smith JC, Bennett S, Evans LM, . The effects of induced hypogonadism on arterial stiffness, body composition, and metabolic parameters in males with prostate cancer. J Clin Endocrinol Metab. 2001;86(9):4261-4267. doi:10.1210/jcem.86.9.7851 11549659

[7] Keating NL, O’Malley AJ, Smith MR. Diabetes and cardiovascular disease during androgen deprivation therapy for prostate cancer. J Clin Oncol. 2006;24(27):4448-4456. doi:10.1200/JCO.2006.06.2497 16983113

[8] Lin E, Garmo H, Van Hemelrijck M, . Exploring the association between use of gonadotropin releasing hormones agonists and prostate cancer diagnosis per se and diabetes control in men with type 2 diabetes mellitus: a nationwide, population-based cohort study. BMC Cancer. 2021;21(1):1259. doi:10.1186/s12885-021-08941-y 34809595 PMC8607667

[9] Hupe MC, Hammerer P, Ketz M, Kossack N, Colling C, Merseburger AS. Retrospective analysis of patients with prostate cancer initiating GnRH agonists/antagonists therapy using a German claims database: epidemiological and patient outcomes. Front Oncol. 2018;8:543. doi:10.3389/fonc.2018.00543 30538951 PMC6277700

[10] Davis MK, Rajala JL, Tyldesley S, Pickles T, Virani SA. The prevalence of cardiac risk factors in men with localized prostate cancer undergoing androgen deprivation therapy in British Columbia, Canada. J Oncol. 2015;2015:820403. doi:10.1155/2015/820403 26300918 PMC4537764

[11] Smith MR, Lee H, McGovern F, . Metabolic changes during gonadotropin-releasing hormone agonist therapy for prostate cancer: differences from the classic metabolic syndrome. Cancer. 2008;112(10):2188-2194. doi:10.1002/cncr.23440 18348297 PMC2562782

[12] Saylor PJ, Smith MR. Metabolic complications of androgen deprivation therapy for prostate cancer. J Urol. 2009;181(5):1998-2006. doi:10.1016/j.juro.2009.01.047 19286225 PMC2900631

[13] Gudbjörnsdottir S, Cederholm J, Nilsson PM, Eliasson B; Steering Committee of the Swedish National Diabetes Register. The National Diabetes Register in Sweden: an implementation of the St. Vincent declaration for quality improvement in diabetes care. Diabetes Care. 2003;26(4):1270-1276. doi:10.2337/diacare.26.4.1270 12663609

[14] Van Hemelrijck M, Adolfsson J, Garmo H, . Risk of thromboembolic diseases in men with prostate cancer: results from the population-based PCBaSe Sweden. Lancet Oncol. 2010;11(5):450-458. doi:10.1016/S1470-2045(10)70038-3 20395174 PMC2861771

[15] George G, Garmo H, Rudman S, . Long-term adherence to GnRH agonists in men with prostate cancer: a nation-wide population-based study in prostate cancer data base Sweden. Scand J Urol. 2020;54(1):20-26. doi:10.1080/21681805.2019.1702093 31842658

[16] Vickers AJ, Assel MJ, Sjoberg DD, . Guidelines for reporting of figures and tables for clinical research in urology. J Urol. 2020;204(1):121-133. doi:10.1097/JU.0000000000001096 32441187

[17] Wasserstein RL, Lazar NA. The ASA statement on P values: context, process, and purpose. Am Stat. 2016;70(2):129-133. doi:10.1080/00031305.2016.1154108

[18] Hayes-Larson E, Kezios KL, Mooney SJ, Lovasi G. Who is in this study, anyway? guidelines for a useful Table 1. J Clin Epidemiol. 2019;114:125-132. doi:10.1016/j.jclinepi.2019.06.011 31229583 PMC6773463

[19] Mohler JL, Antonarakis ES, Armstrong AJ, . Prostate cancer, version 2.2019, NCCN clinical practice guidelines in oncology. J Natl Compr Canc Netw. 2019;17(5):479-505. doi:10.6004/jnccn.2019.0023 31085757

[20] Zethelius B, Eliasson B, Eeg-Olofsson K, Svensson A-M, Gudbjörnsdottir S, Cederholm J; NDR. A new model for 5-year risk of cardiovascular disease in type 2 diabetes, from the Swedish National Diabetes Register (NDR). Diabetes Res Clin Pract. 2011;93(2):276-284. doi:10.1016/j.diabres.2011.05.037 21719139

[21] Swedish National Diabetes Register. Nationwide results: 1996-2019. Accessed July 7, 2022. https://www.ndr.nu/pdfs/NationWideResults_1996-2019.pdf

[22] Williams B, Mancia G, Spiering W, ; ESC Scientific Document Group. 2018 ESC/ESH Guidelines for the management of arterial hypertension. Eur Heart J. 2018;39(33):3021-3104. doi:10.1093/eurheartj/ehy339 30165516

[23] Salmon C, Song L, Muir K, ; UKGPCS Collaborators; APCB BioResource (Australian Prostate Cancer BioResource); on behalf of the PRACTICAL Consortium. Marital status and prostate cancer incidence: a pooled analysis of 12 case-control studies from the PRACTICAL consortium. Eur J Epidemiol. 2021;36(9):913-925. doi:10.1007/s10654-021-00781-1 34275018

[24] George G, Rudman S, Fleure L, . Qualitative analysis of interviews and focus groups exploring factors contributing to adherence to GnRH agonists in men with prostate cancer. Semin Oncol Nurs. Published online December 14, 2021. doi:10.1016/j.soncn.2021.151236 34920915

[25] Dhindsa DS, Khambhati J, Schultz WM, Tahhan AS, Quyyumi AA. Marital status and outcomes in patients with cardiovascular disease. Trends Cardiovasc Med. 2020;30(4):215-220. doi:10.1016/j.tcm.2019.05.012 31204239

[26] Khaing W, Vallibhakara SA, Attia J, McEvoy M, Thakkinstian A. Effects of education and income on cardiovascular outcomes: a systematic review and meta-analysis. Eur J Prev Cardiol. 2017;24(10):1032-1042. doi:10.1177/2047487317705916 28406328

[27] Lund Nilsen TI, Johnsen R, Vatten LJ. Socio-economic and lifestyle factors associated with the risk of prostate cancer. Br J Cancer. 2000;82(7):1358-1363. doi:10.1054/bjoc.1999.1105 10755415 PMC2374496

[28] Tsai HK, D’Amico AV, Sadetsky N, Chen M-H, Carroll PR. Androgen deprivation therapy for localized prostate cancer and the risk of cardiovascular mortality. J Natl Cancer Inst. 2007;99(20):1516-1524. doi:10.1093/jnci/djm168 17925537

[29] D’Amico AV, Denham JW, Crook J, . Influence of androgen suppression therapy for prostate cancer on the frequency and timing of fatal myocardial infarctions. J Clin Oncol. 2007;25(17):2420-2425. doi:10.1200/JCO.2006.09.3369 17557956

[30] Levine GN, D’Amico AV, Berger P, ; American Heart Association Council on Clinical Cardiology and Council on Epidemiology and Prevention, the American Cancer Society, and the American Urological Association. Androgen-deprivation therapy in prostate cancer and cardiovascular risk: a science advisory from the American Heart Association, American Cancer Society, and American Urological Association: endorsed by the American Society for Radiation Oncology. CA Cancer J Clin. 2010;60(3):194-201. doi:10.3322/caac.20061 20124400 PMC3049943

[31] Nguyen PL, Je Y, Schutz FA, . Association of androgen deprivation therapy with cardiovascular death in patients with prostate cancer: a meta-analysis of randomized trials. JAMA. 2011;306(21):2359-2366. doi:10.1001/jama.2011.1745 22147380

[32] Bosco C, Bosnyak Z, Malmberg A, Adolfsson J, Keating NL, Van Hemelrijck M. Quantifying observational evidence for risk of fatal and nonfatal cardiovascular disease following androgen deprivation therapy for prostate cancer: a meta-analysis. Eur Urol. 2015;68(3):386-396. doi:10.1016/j.eururo.2014.11.039 25484142

[33] Meng F, Zhu S, Zhao J, . Stroke related to androgen deprivation therapy for prostate cancer: a meta-analysis and systematic review. BMC Cancer. 2016;16(1):180. doi:10.1186/s12885-016-2221-5 26940836 PMC4778362

[34] Jin C, Fan Y, Meng Y, . A meta-analysis of cardiovascular events in intermittent androgen-deprivation therapy versus continuous androgen-deprivation therapy for prostate cancer patients. Prostate Cancer Prostatic Dis. 2016;19(4):333-339. doi:10.1038/pcan.2016.35 27595915

[35] Crawford ED, Schally AV, Pinthus JH, . The potential role of follicle-stimulating hormone in the cardiovascular, metabolic, skeletal, and cognitive effects associated with androgen deprivation therapy. Urol Oncol. 2017;35(5):183-191. doi:10.1016/j.urolonc.2017.01.025 28325650

[36] Saylor PJ, Keating NL, Freedland SJ, Smith MR. Gonadotropin-releasing hormone agonists and the risks of type 2 diabetes and cardiovascular disease in men with prostate cancer. Drugs. 2011;71(3):255-261. doi:10.2165/11588930-000000000-00000 21319864 PMC3671348

[37] Rydén L, Standl E, Bartnik M, ; Task Force on Diabetes and Cardiovascular Diseases of the European Society of Cardiology (ESC); European Association for the Study of Diabetes (EASD). Guidelines on diabetes, pre-diabetes, and cardiovascular diseases: executive summary. Eur Heart J. 2007;28(1):88-136. doi:10.1016/S1885-5857(07)60205-9 17220161

[38] Qu M, Feng C, Wang X, . Association of serum testosterone and luteinizing hormone with blood pressure and risk of cardiovascular disease in middle-aged and elderly men. J Am Heart Assoc. 2021;10(7):e019559. doi:10.1161/JAHA.120.019559 33739129 PMC8174322

[39] Morgans AK, Shore N, Cope D, . Androgen receptor inhibitor treatments: cardiovascular adverse events and comorbidity considerations in patients with non-metastatic prostate cancer. Urol Oncol. 2021;39(1):52-62. doi:10.1016/j.urolonc.2020.08.003 32958445

[40] Rezaei MM, Rezaei MM, Ghoreifi A, Kerigh BF. Metabolic syndrome in patients with prostate cancer undergoing intermittent androgen-deprivation therapy. Can Urol Assoc J. 2016;10(9-10):E300-E305. doi:10.5489/cuaj.3655 27695584 PMC5028214

[41] Kienitz T, Quinkler M. Testosterone and blood pressure regulation. Kidney Blood Press Res. 2008;31(2):71-79. doi:10.1159/000119417 18319594

[42] Dziopa K, Asselbergs FW, Gratton J, Chaturvedi N, Schmidt AF. Cardiovascular risk prediction in type 2 diabetes: a comparison of 22 risk scores in primary care settings. Diabetologia. 2022;65(4):644-656. doi:10.1007/s00125-021-05640-y 35032176 PMC8894164

