# Supplementary material for: Association of Gonadotropin-Releasing Hormone Agonists for Prostate Cancer With Cardiovascular Disease Risk and Hypertension in Men With Diabetes
Source: JAMA Netw Open. 2022 Aug 8;5(8):e2225600. doi: 10.1001/jamanetworkopen.2022.25600 (PMC9361086; doi:10.1001/jamanetworkopen.2022.25600)
Supplement: Supplement. — eTable 1. HR and 95% CI for Worsening Hypertension Using Different Definitions of Events in PCa Exposure Cohort and GnRH Exposure Cohort: Crude Model eTable 2. Number of Events in PCa Cohort and GnRH Cohort eFigure 1. Patient Inclusion and Exclusion Flowchart eFigure 2. Schematic Diagram for the Selection Process of Exposed Men and Nonexposed Men in NDR Registrations eFigure 3. Blood Pressure Levels Change Over Time by PCa Status in PCa Exposure Cohort eFigure 4. Blood Pressure Levels Change Over Time by PCa Status in GnRH Exposure Cohorts [file jamanetwopen-e2225600-s001.pdf]

Supplemental Online Content

Lin E, Garmo H, Van Hemelrijck M, et al. Association of gonadotropin-releasing hormone agonists for prostate cancer with cardiovascular disease risk and hypertension in men with diabetes. *JAMA Netw Open.* 2022;5(8):e2225600. doi:10.1001/jamanetworkopen.2022.25600

eTable 1. HR and 95% CI for Worsening Hypertension Using Different Definitions of Events in PCa Exposure Cohort and GnRH Exposure Cohort: Crude Model

eTable 2. Number of Events in PCa Cohort and GnRH Cohort

eFigure 1. Patient Inclusion and Exclusion Flowchart

eFigure 2. Schematic Diagram for the Selection Process of Exposed Men and Nonexposed Men in NDR Registrations

eFigure 3. Blood Pressure Levels Change Over Time by PCa Status in PCa Exposure Cohort

eFigure 4. Blood Pressure Levels Change Over Time by PCa Status in GnRH Exposure Cohorts

This supplemental material has been provided by the authors to give readers additional information about their work.

© 2022 Lin E et al. *JAMA Network Open.*

eTable 1. HR and 95% CI for Worsening Hypertension Using Different Definitions of Events in PCa Exposure Cohort and GnRH Exposure Cohort: Crude Model

Exposure	BP rose to 140/80 (<= 65-year-old) or 130/80 mmHg (>65-year-old) [a]	SBP increased 10mmHg [b]	DBP increased 5mmHg [c]	Number of Anti-hypertension changes [d]	Dosage of anti-hypertension changes [d]
	HR (95% CI)	HR (95% CI)	HR (95% CI)	HR (95% CI)	HR (95% CI)
PCa exposure cohort					
**Using GnRH agonists**					
No PCa	Ref.	Ref.	Ref.	Ref.	Ref.
PCa without GnRH	0.89 (0.83-0.95)	0.92 (0.88-0.97)	0.87 (0.83-0.92)	0.98 (0.93-1.03)	0.99 (0.95-1.04)
PCa with GnRH	0.68 (0.60-0.77)	0.80 (0.72-0.87)	0.76 (0.70-0.83)	0.94 (0.85-1.03)	0.98 (0.90-1.07)
**PCa diagnosis**					
No	Ref.	Ref.	Ref.	Ref.	Ref.
Yes	0.83 (0.78-0.89)	0.89 (0.85-0.93)	0.85 (0.81-0.88)	0.97 (0.92-1.02)	1.00 (0.95-1.04)
**PCa risk category**					
No PCa	Ref.	Ref.	Ref.	Ref.	Ref.
Low-risk	1.08 (0.95-1.22)	0.96 (0.88-1.04)	0.91 (0.83-0.99)	1.00 (0.91-1.10)	0.99 (0.91-1.08)
Intermediate-risk	0.85 (0.76-0.95)	0.88 (0.82-0.95)	0.86 (0.80-0.93)	1.05 (0.97-1.13)	1.06 (0.98-1.13)
High-risk	0.77 (0.68-0.86)	0.83 (0.77-0.91)	0.78 (0.72-0.85)	0.95 (0.87-1.04)	1.00 (0.92-1.08)
Regional metastasises	0.65 (0.50-0.85)	0.90 (0.76-1.06)	0.76 (0.64-0.90)	0.81 (0.67-0.98)	0.86 (0.72-1.02)
Distance metastasises	0.64 (0.52-0.78)	0.89 (0.78-1.02)	0.86 (0.75-0.98)	0.76 (0.64-0.89)	0.78 (0.67-0.91)
Missing data	0.88 (0.64-1.22)	1.01 (0.81-1.26)	0.97 (0.78-1.20)	0.98 (0.77-1.25)	1.09 (0.88-1.35)
GnRH exposure cohort					
**Using GnRH agonists**					
PCa without GnRH	Ref.	Ref.	Ref.	Ref.	Ref.
PCa with GnRH	0.69 (0.57-0.84)	0.93 (0.82-1.07)	0.98 (0.86-1.11)	1.04 (0.90-1.19)	1.04 (0.90-1.19)
**PCa risk category**					

© 2022 Lin E et al. *JAMA Network Open.*

Low-risk	Ref.	Ref.	Ref.	Ref.	Ref.
**Exposure**	**BP rose to 140/80 (<= 65-year-old) or 130/80 mmHg (>65-year-old) [a]**	**SBP increased 10mmHg [b]**	**DBP increased 5mmHg [c]**	**Number of Anti-hypertension changes [d]**	**Dosage of anti-hypertension changes [d]**
	**HR (95% CI)**	**HR (95% CI)**	**HR (95% CI)**	**HR (95% CI)**	**HR (95% CI)**
Intermediate-risk	0.97 (0.83-1.14)	0.98 (0.88-1.09)	1.01 (0.91-1.12)	1.04 (0.93-1.16)	1.04 (0.93-1.16)
High-risk	0.89 (0.74-1.07)	0.84 (0.73-0.96)	1.03 (0.90-1.17)	1.04 (0.90-1.19)	1.04 (0.90-1.19)
Regional metastasises	0.82 (0.49-1.38)	0.64 (0.45-0.92)	0.64 (0.45-0.92)	1.16 (0.85-1.60)	1.16 (0.85-1.60)
Distance metastasises	0.85 (0.48-1.52)	0.49 (0.30-0.80)	0.85 (0.57-1.25)	0.75 (0.48-1.18)	0.75 (0.48-1.18)
Missing data	0.87 (0.56-1.35)	0.90 (0.68-1.19)	0.84 (0.63-1.12)	1.16 (0.87-1.54)	1.16 (0.87-1.54)

a. We excluded those men with missing data on SBP and DBP.
b. We excluded those men with missing data on SBP.
c. Those men with missing data on DBP were excluded.
d. Those men with missing data on antihypertensive drugs were excluded.

Abbreviations: HR= Hazard ratio; 95% CI= 95% confidence interval; PCa=prostate cancer; GnRH= Gonadotropin-releasing hormone agonists; CVD= cardiovascular diseases; BP=blood pressure; SBP= systolic blood pressure; DBP= diastolic blood pressure.

© 2022 Lin E et al. *JAMA Network Open.*

eTable 2. Number of Events in PCa Cohort and GnRH Cohort

	PCa cohort		GnRH cohort	
	**men with PCa on/not on GnRH No.=**5,714	**PCa-free men No.=**28,445	**men with PCa on GnRH No.=**692	**men with PCa no on GnRH No.=**3,460
	**No. (%)**	**No. (%)**	**No. (%)**	**No. (%)**
**10% increase in CVD risk**				
No	1,263 (22.1)	4,967 (17.5)	386 (55.8)	2,087 (60.3)
Yes	4,451 (77.9)	23,478 (82.5)	306 (44.2)	1,373 (39.7)
Total number of patients in the subset	5714 (100.0)	28,445 (100.0)	692 (100.0)	3,460 (100.0)
**5% increase in CVD risk**				
No	2,096 (36.7)	9,928 (34.9)	285 (41.2)	1,385 (40.0)
Yes	3,618 (63.3)	18,517 (65.1)	407 (58.8)	2,075 (60.0)
Total number of patients in the subset	5714 (100.0)	28,445 (100.0)	692 (100.0)	3,460 (100.0)
**BP rose by 140 or 130/80mmHg or higher**				
No	1,060 (51.7)	4,733 (45.5)	205 (63.9)	638 (49.3)
Yes	989 (48.3)	5,678 (54.5)	116 (36.1)	656 (50.7)
Total number of patients in the subset	2,049 (100.0)	10,411 (100.0)	321 (100.0)	1,294 (100.0)
**SBP increased 10mmHg**				
No	3,300 (58.8)	14,952 (53.7)	423 (62.9)	1,897 (56.3)
Yes	2,314 (41.2)	12,878 (46.3)	250 (37.1)	1,475 (43.7)
Total number of patients in the subset	5,614 (100.0)	27,830 (100.0)	673 (100.0)	3,372 (100.0)
**DBP increased 5mmHg**				
No	3,279 (58.5)	14,427 (51.9)	400 (59.7)	1,816 (53.9)
Yes	2,326 (41.5)	13,370 (48.1)	270 (40.3)	1,556 (46.1)
Total number of patients in the subset	5,605 (100.0)	27,797 (100.0)	670 (100.0)	3,372 (100.0)
**Number of antihypertension drugs change**				
No	3,710 (64.9)	17,839 (61.7)	498 (72.0)	2,278 (65.8)
Yes	2,004 (35.1)	11,060 (38.3)	194 (28.0)	1,182 (34.2)
Total number of patients in the subset	5,714 (100.0)	28,899 (100.0)	692 (100.0)	3,460 (100.0)
**Dosage of antihypertension drugs change**				
No	3,351 (58.7)	16,066 (56.5)	451 (65.2)	2,104 (60.8)
Yes	2,362 (41.3)	12,379 (43.5)	241 (34.8)	1,356 (39.2)
Total number of patients in the subset	5,713 (100.0)	28,445 (100.0)	692 (100.0)	3,460 (100.0)

Abbreviations: PCa=prostate cancer; GnRH= Gonadotropin-releasing hormone agonists; CVD= cardiovascular diseases; BP=blood pressure; SBP= systolic blood pressure; DBP= diastolic blood pressure.

© 2022 Lin E et al. *JAMA Network Open.*

eFigure 1. Patient Inclusion and Exclusion Flowchart

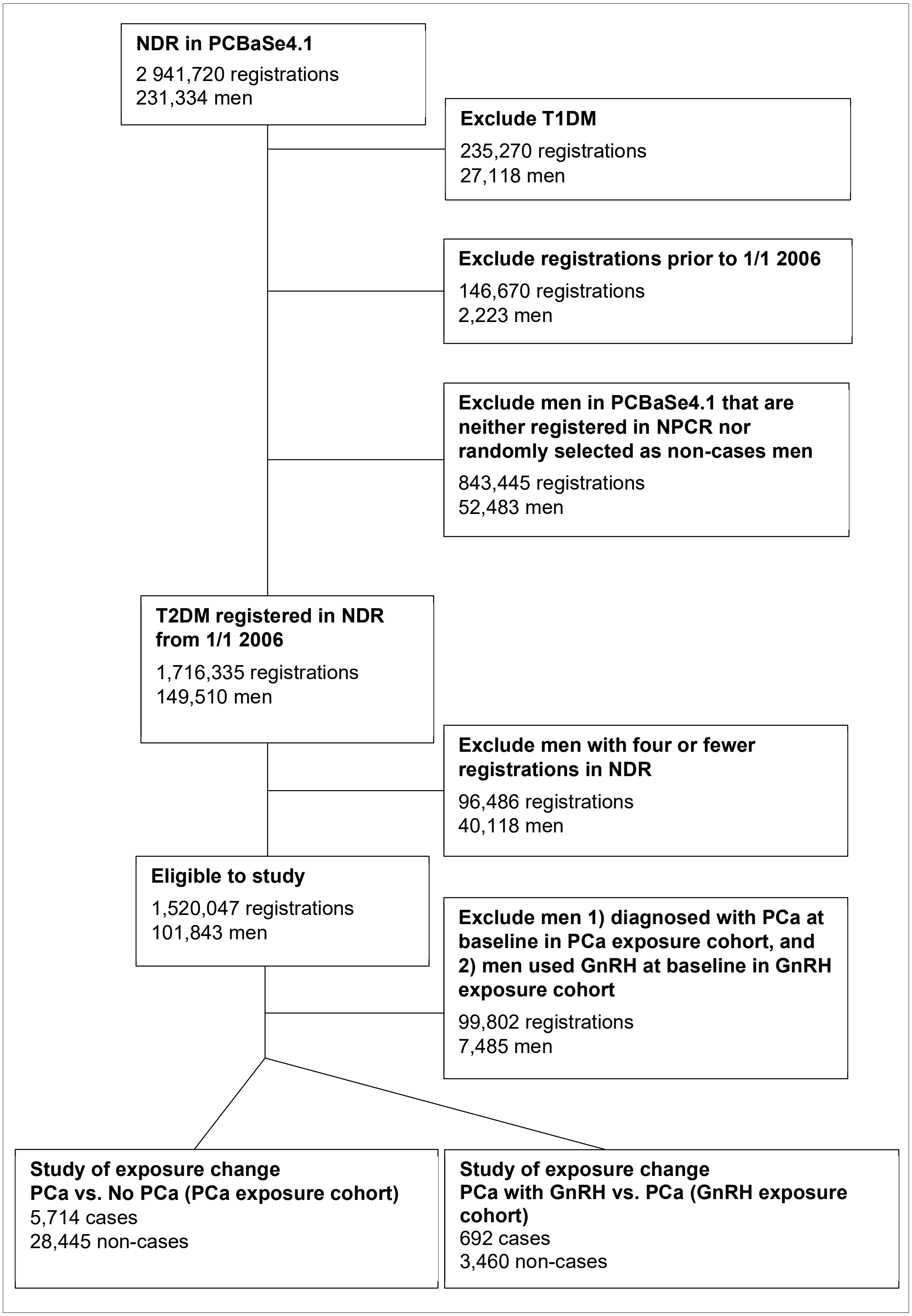

© 2022 Lin E et al. *JAMA Network Open.*

eFigure 2. This figure illustrated the study design and patient selection process. We included men diagnosed with type 2 diabetes mellitus (T2DM), according to the National Diabetes Registry (NDR), amongst men included in Prostate Cancer data Base Sweden (PCBaSe) 4.1 in 2006-2016 and created two cohorts – "Prostate cancer (PCa) + Gonadotropin-releasing hormone agonists (GnRH) exposure cohort" and "GnRH exposure cohort". 5,714 men with PCa and 28,445 PCa-free men were included in the PCa+ exposure cohort. The GnRH exposure cohort contained 692 men with PCa who started GnRH after PCa diagnosis and 3,460 men with PCa not using GnRH as non-cases.
Abbreviations: PCa=prostate cancer; GnRH= Gonadotropin-releasing hormone agonists; NDR= the National Diabetes Register; PCBaSe = Prostate Cancer data Base Sweden.

© 2022 Lin E et al. *JAMA Network Open.*

eFigure 2. Schematic Diagram for the Selection Process of Exposed Men and Nonexposed Men in NDR Registrations

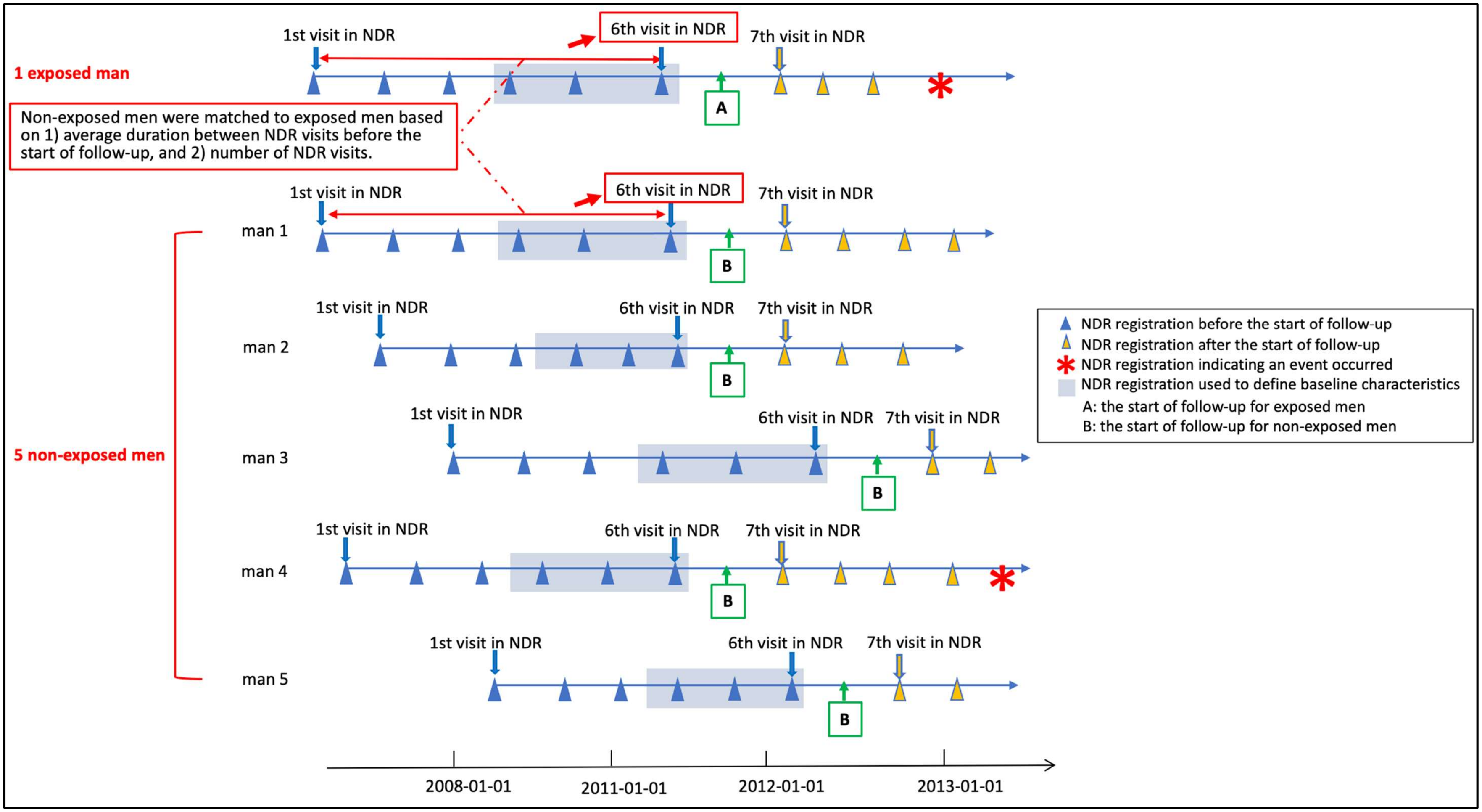

Abbreviations: NDR= the National Diabetes Register

© 2022 Lin E et al. *JAMA Network Open.*

eFigure 3. Blood Pressure Levels Change Over Time by PCa Status in PCa Exposure Cohort

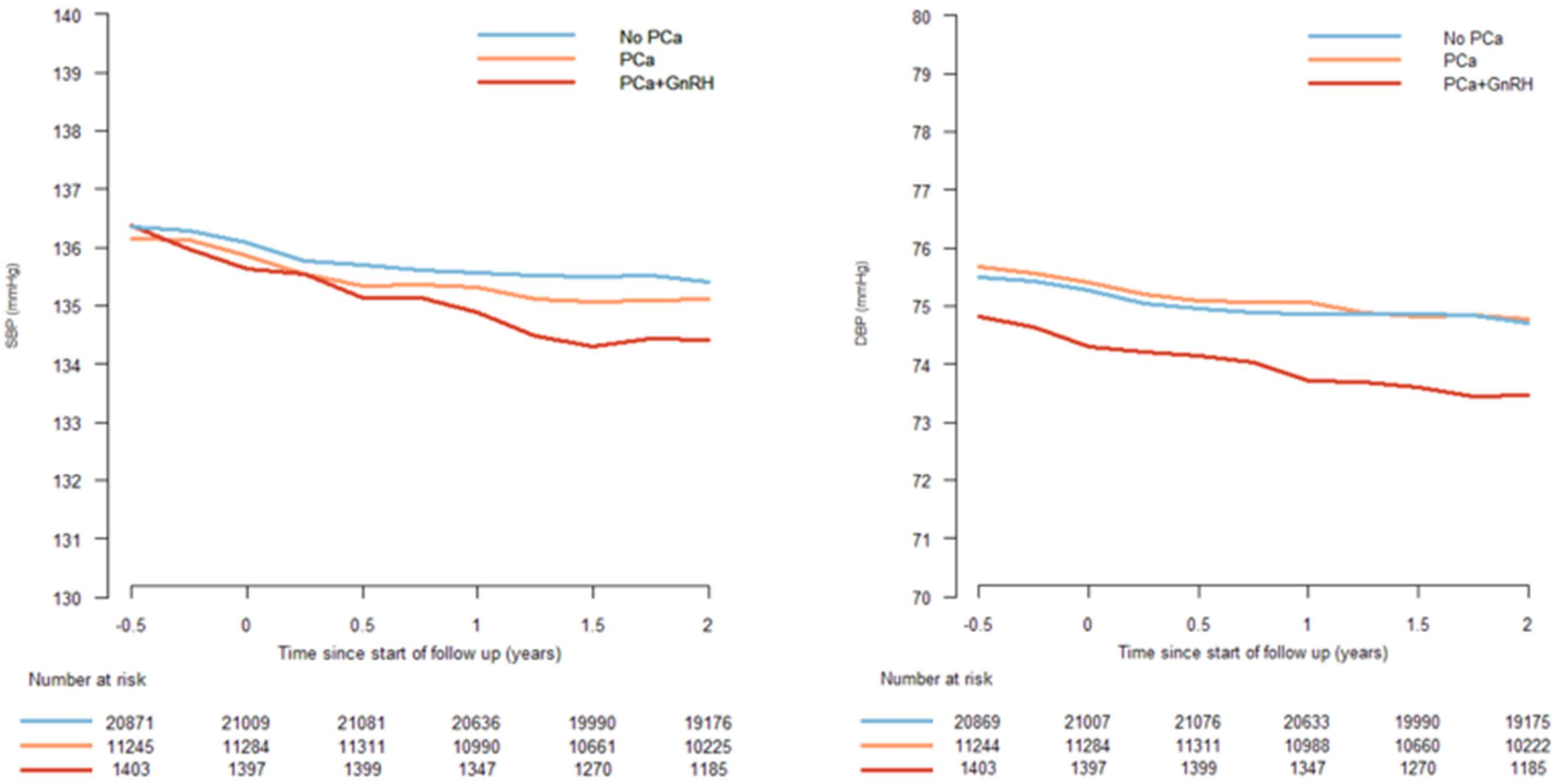

In eFigure 3, we found that blood pressure levels in men with PCa with GnRH were lower than men without GnRH over time (eFigure 3).

Abbreviations: PCa= prostate cancer; GnRH= Gonadotropin-releasing hormone agonists; BP=blood pressure; SBP= systolic blood pressure; DBP= diastolic blood pressure.

© 2022 Lin E et al. *JAMA Network Open.*

eFigure 4. Blood Pressure Levels Change Over Time by PCa Status in GnRH Exposure Cohort

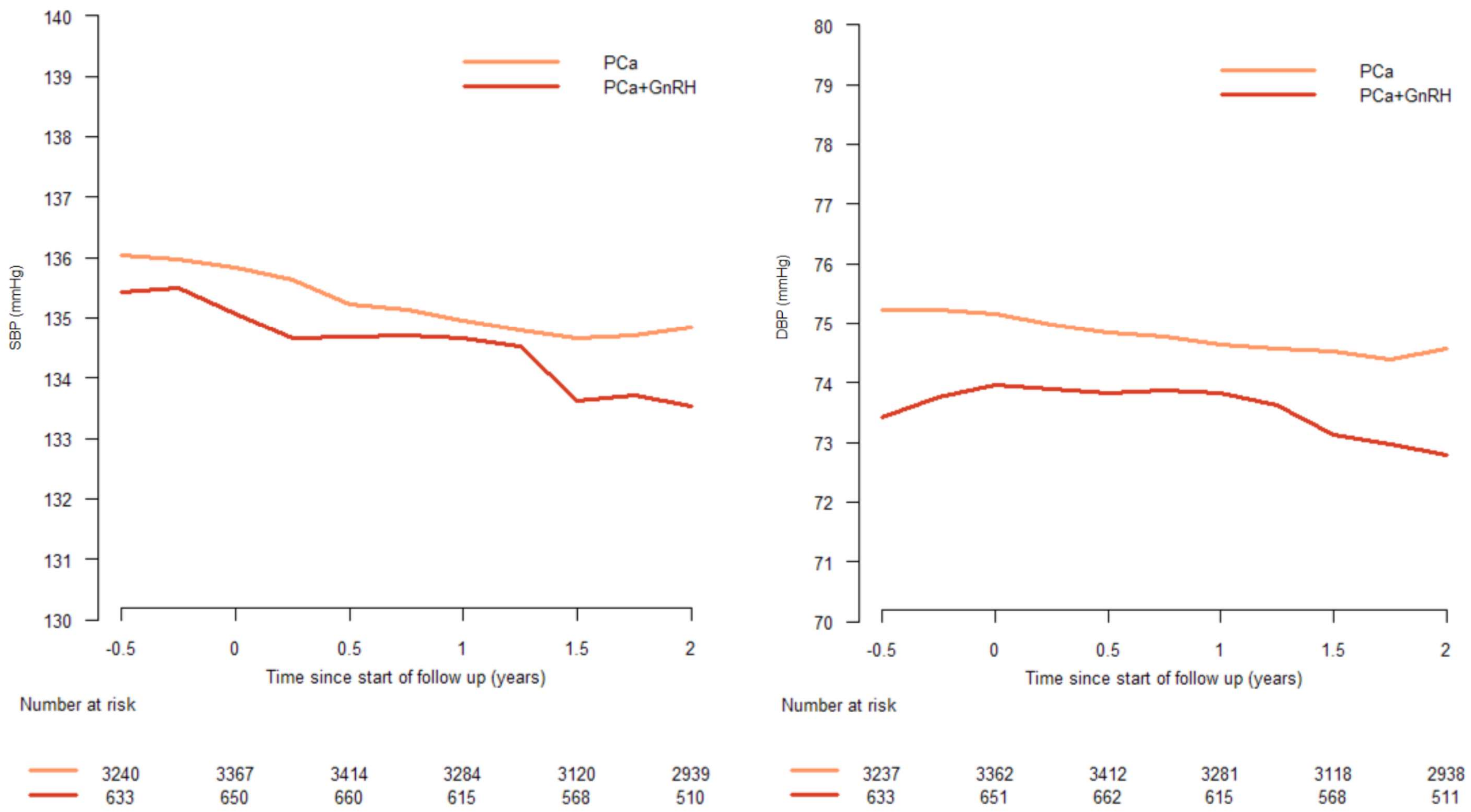

The SBP and DBP in these men were always lower than for men with PCa without GnRH over time (eFigure 4).
Abbreviations: PCa= prostate cancer; GnRH= Gonadotropin-releasing hormone agonists; BP=blood pressure; SBP= systolic blood pressure; DBP= diastolic blood pressure.

© 2022 Lin E et al. *JAMA Network Open.*
